# Supplementary figures and images for: A diverse global fungal library for drug discovery
Source: PeerJ. 2020 Nov 27;8:e10392. doi: 10.7717/peerj.10392 (PMC7703384; doi:10.7717/peerj.10392)

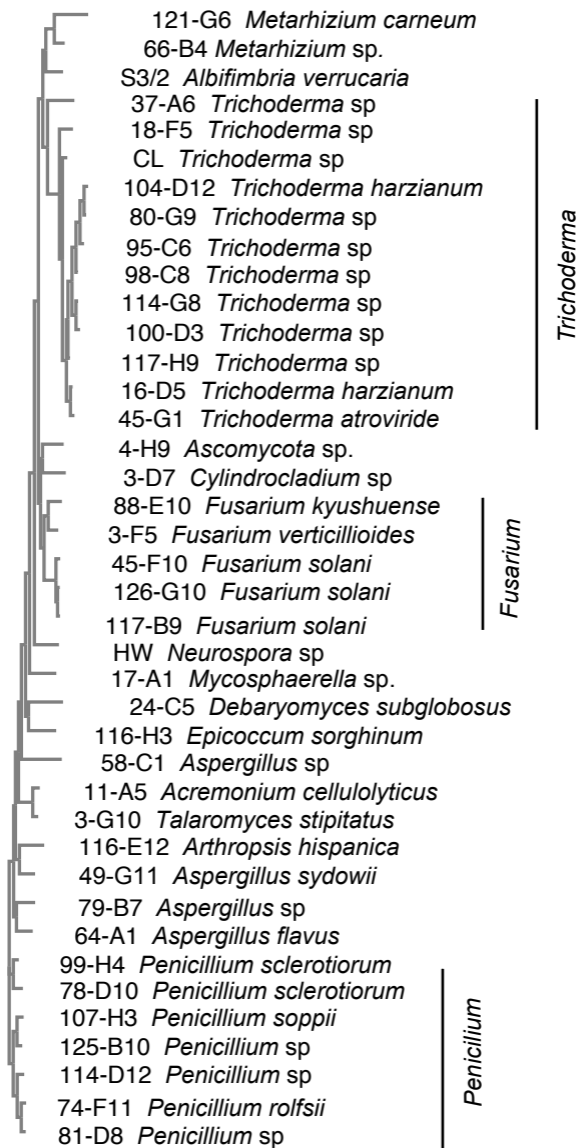

Supplement: Supplemental Information 1 — The phylogenetic tree was constructed based on their ITS sequences. Three large genus groups predominantly presented in soils and associated with plants were labeled. [file peerj-08-10392-s001.pdf]
